# Effect of vitamin B6 on pain, disease severity, and psychological profile of fibromyalgia patients; a randomized, double-blinded clinical trial

**DOI:** 10.1186/s12891-022-05637-7

**Published:** 2022-07-13

**Authors:** Banafsheh Ghavidel-Parsa, Arvin Naeimi, Faeze Gharibpoor, Nazila Sattari, Atefeh Jafari, Irandokht Shenavar Masooleh, Ali Montazeri

**Affiliations:** 1 Rheumatology Research Center, Razi Hospital, School of Medicine, Guilan University of Medical Science, Rasht, Iran; 2 Student Research Committee, Deputy of Research and Technology, Guilan University of Medical Sciences, Namjoo St, Rasht, 41446-66949 Guilan Iran; 3 Department of Clinical Pharmacy, School of Pharmacy, Guilan University of Medical Sciences, Rasht, Iran; 4 Population Health Research Group, Health Metrics Research Center, Iranian Institute for Health Sciences Research, ACECR, Tehran, Iran

**Keywords:** Fibromyalgia, Pain, Vitamin B6, Pyridoxine

## Abstract

**Background:**

Given the role of vitamin B6 on pronociceptive/antinociceptive neurotransmitters balance, metabolic reactions, and inflammation, it is important to clarify the effect of vitamin B6 on pain and psychological disturbance in fibromyalgia (FM). This study aimed to evaluate whether an 80-mg daily dose of vitamin B6 improves pain, disease severity and psychological symptoms of FM compared to a placebo.

**Methods:**

This randomized, double-blinded, placebo-controlled trial was performed on the FM patients whose diagnosis was confirmed by a rheumatologist based on the 2016 American College of Rheumatology (ACR). 90 Patients were randomized to receive either vitamin B6 (80 mg daily) or placebo in a 1:1 ratio, with a permuted block size of 30 stratified by disease severity. Primary outcomes included the Revised Fibromyalgia Impact Questionnaire (FIQR), Hospital Anxiety and Depression Scale (HADS), 12-item short-form health survey (SF-12), and pain visual analog scale (pain-VAS)). The mean differences in outcomes (before and after treatment) were compared between the vitamin B6 and placebo groups using an independent T-test. An ANCOVA model adjusted for baseline outcome value was also provided to compare the outcomes between the two groups.

**Results:**

Of 90 eligible patients, 60 patients (31 patients in vitamin B6 and 29 in the placebo group) completed the trial. Overall, the FIQR, pain-VAS, and HADS-anxiety scores improved after treatment in both vitamin B6 and placebo groups; However, there was no statistically significant intergroup difference regarding primary outcomes. ANCOVA model also showed no difference in the treatment effects.

**Conclusions:**

Our results showed no priority for vitamin B6 over placebo in FM patients. Considering the potential ameliorating role of vitamin B6 on pain and psychological symptoms, acknowledgment of vitamin B6 as a relatively safe adjuvant treatment needs larger future studies.

**Trial registration:**

Iranian Registry of Clinical Trials: IRCT20200920048782N2 on 2021/10/04.

## Introduction

Fibromyalgia (FM) has been characterized by widespread chronic pain, fatigue, sleep disturbances, cognitive dysfunction, and polysymptomatic complaints [1]. It is a common and debilitating chronic nociplastic pain disorder representing significant diagnostic and therapeutic challenges. The continuing difficulties in treatment may derive from the unclear and heterogeneous pathophysiological basis displaying diverse FM phenotypes [2]. However, the central pain sensitization, as the denouement of various pathophysiologic pathways, has been purported as the primary pathogenesis of FM [3].

Neurobiological studies of central sensitization show evidence of neurochemical imbalances between pronociceptive and antinociceptive brain areas in FM patients. High levels of excitatory neurotransmitters of glutamate and substance P and reduced levels of serotonin, norepinephrine, and γ-aminobutyric acid (GABA) have been found in FM patients [2, 4]. In addition, immune system abnormalities have also been demonstrated in individuals with central sensitization [5]. A growing body of evidence proposes the role of autoimmunity and low-grade inflammation as the epicenter of FM pathogenesis. In this line, the altered level of platelet cell distribution width (PDW), mean platelet volume (MPV), and neutrophil leukocyte ratio (NLR) reported in FM supports the inflammation theory of FM [6, 7].

Vitamin B6, as an essential cofactor in the metabolic reactions of homocysteine, regulates the transformation of various neurotransmitters such as GABA, epinephrine, serotonin, and melatonin which are involved in the diverse behavioral processes, including sleep, learning, memory, and sensation of pain [8, 9]. Thereby, due to the potential effect of vitamin B6 on neuronal plasticity, it would be conceivable if it is implicated as a pain or mood modifier. Indeed, several endeavors have been performed to demonstrate the analgesic and mood-regulating effects of vitamin B6 in different patient groups, including carpal tunnel syndromes, diabetic neuropathy, back pain, depression, and migraine headache [10–12]. In addition, preclinical studies support the pain modulator effect of vitamin B6 in rats. Vitamin B6 partially attenuates thalamic evoked nociceptive burst discharge and alleviates mechanical allodynia in diabetic rats [10, 13]. Furthermore, some reports show the anti-inflammatory and anti-oxidant effects of vitamin B6 in inflammatory conditions. Vitamin B6 supplementation has lowered serum levels of IL-6 and TNF-α in patients with rheumatoid arthritis and has been demonstrated to suppress IL-1β productions [14, 15].

Despite the apparent potential benefit of vitamin B6 on pain and depression, no research has investigated the clinical implication of vitamin B6 on the pain or psychological profile of FM patients. FM, as the prototypical nociplastic pain condition, shows the aforementioned neurotransmitter imbalance and mild inflammatory status that can be targeted by vitamin B6 treatment. Given FM as a difficult therapeutic condition and the relative safety of vitamin B6 treatment, it would be reasonable to evaluate the effect of vitamin B6 on pain, FM impact, and health status of these patients. In this study, we aimed to assess the effect of the administration of vitamin B6 on the physical pain, disease severity, and psychological symptoms of FM patients compared to placebo groups. Our secondary objective was to evaluate whether vitamin B6, as an anti-inflammatory agent, could change the status of inflammatory markers in FM patients.

## Material and methods

### Study design, setting, and participants

This study was a randomized, double-blinded, placebo-controlled, parallel-group trial on 90 FM patients admitted to the FM clinic of Razi academic hospital affiliated with Guilan University of Medical Sciences (GUMS) from October 2021 to January 2022. The diagnosis of FM was made by one rheumatologist (B.GH) according to the 2016 American College of Rheumatology (2016 ACR) criteria [16]. Patients were excluded if they were under 18 years old, pregnant or breastfeeding, suffering from any comorbidity with nociceptive or neuropathic chronic pain or inflammation (e.g., major recent trauma, malignancy, other non-inflammatory or inflammatory rheumatic diseases, inflammatory bowel disease, and any kind of neuropathic pain).

The study protocol was approved by the ethics committee of Guilan University of Medical Sciences (IR.GUMS.REC.1400.258) in accordance with the World Medical Association's code of ethics (Declaration of Helsinki, revised in Brazil 2013) and registered at an ICMJE and WHO recognized registry of clinical trials (Iranian Registry of Clinical Trials: www.irct.ir) on 2021/10/04 (registration number: IRCT20200920048782N2). All participants were informed about the study design and then written informed consent was obtained from all volunteers. They were informed that their level of care wouldn't be affected if they discontinued the study.

### Randomization and blinding

Using stratified randomization, three strata based on the disease severity, which were obtained from the Revised Fibromyalgia Impact Questionnaire (RFIQ) (mild:0–39, moderate: 40–59, severe: 60–100), were formed. Thereafter, for each stratum, randomization in the block size of 30 was performed. The allocation ratio for each block was 1:1, and patients were randomized to the vitamin B6 or placebo groups. A random sequence for each block was generated using the Randomization.com website. A research staff, who wasn't involved in data collection and data entry, was in charge of generating randomized sequence and allocation concealment. For allocation concealment, the sequentially numbered, sealed, opaque envelopes were used. Active drug and placebo were kept in completely identical (in shape, color, size, and taste) plum bottles, codded as either A or B. Group allocation was unknown to the investigators, data collectors, and patients. Three medical students were responsible for enrolling eligible patients, calculating disease severity, and assigning them to either receive vitamin B6 or a placebo according to the randomized sequence.

### Intervention

At the initial visit, patients were evaluated for demographic data (including age, marital status, education level, occupation, time since their first symptoms, time to a confirmed diagnosis, and prior medications) and outcome measures. A secondary visit at day 60 ± 3 was made to re-evaluate the outcome measures.

Primary outcome measures included the Revised Fibromyalgia Impact Questionnaire (FIQR), Hospital Anxiety and Depression Scale (HADS), 12-item short-form health survey (SF-12), and pain visual analog scale (pain-VAS). Three medical students helped patients to fulfill the questionnaires.

Secondary outcomes were defined as platelet distribution width (PDW), mean platelet volume (MPV), red blood cell distribution width (RDW), and neutrophil leukocyte ratio (NLR).

Patients were instructed to take either vitamin B6 or a placebo twice daily for 60 days. The intervention group received the pills of vitamin B6 (40 mg, Iran hormone company), and the placebo group received pills with the same shape as vitamin B6 (containing lactose monohydrate (78%), corn starch (20%), aerosil (0.5%), talc (1%) and magnesium stearate (0.5%), produced under standard condition by Pharmacology faculty of Guilan University of Medical Sciences).

Patients taking prior medication to relieve FM symptoms were encouraged to continue their regimen. Patients who were already taking any supplement containing vitamin B6 were asked to stop taking them for a minimum of 2 weeks before the study. An intake calendar was given to patients to track their compliance. The patients were also informed to report any unpredicted symptoms at any time during the study.

### Blood sampling and laboratory assays

Patients were referred to a private laboratory accredited by the health ministry (Razi Pathobiology Laboratory, Rasht, Iran) for blood sampling and laboratory analysis. Blood samples were collected from non-fasting patients at baseline and after 60 ± 3 days. Blood samples were analyzed for complete blood count and differentials and erythrocyte sedimentation rate (ESR). Platelet distribution width (PDW), Mean platelet volume (MPV), red blood cell distribution width (RDW), and Neutrophil leukocyte ratio (NLR) were recorded as secondary outcomes.

### Outcome measures

The FIQR is a 21-items questionnaire with an 11-point numeric rating scale for each question which assesses clinical symptom severity and disease impact in patients with FM [17]. The total score of the FIQR ranges between 0–100, with a higher score indicating worse disease impact. FIQR has three domains evaluating patients' function, overall impact, and symptoms severity. The SF-12 questionnaire evaluates eight domains of the health status, formulating the Mental Component Summary (MCS) and Physical Component Summary (PCS). Scores range from "0 to 100" where "0" indicates the worst condition and "100" indicates the best possible condition [18].

HADS is a 14 items questionnaire with two subclasses: 7 questions for anxiety and 7 questions for depression. Each question scores between 0 to 3, with a maximum score of 21 for each subclass. Higher scores mean a higher level of anxiety and depression [19].

### Sample size calculation

The sample size was calculated using G*Power 3.1 [20]. Since there was no prior study on vitamin B6 in FM, we obtained pain-VAS scores from a previous study by Sadeghi et al., who measured the effect of vitamin B6 on migraine headache [12]. Type 1 error (two-tailed) was considered 5% with 80% power. The calculated effect size was 0.75. The minimum sample size with 30% dropout was 43 patients in each group.

### Statistical analysis

Descriptive statistics were used to calculate the mean, median, and standard deviation for continuous variables and frequency for categorical variables. Shapiro–Wilk test was used to test the normality of data, and Levene's test was used for the equality of variances. To compare before and after treatment effects within each group, we used Paired t-test and Wilcoxon test for normally distributed and non-normally distributed scores' changes, respectively. To compare the outcomes between vitamin B6 and placebo groups, an independent T-test and Mann–Whitney U test were used for parametric and non-parametric data, respectively.

For each outcome, an ANCOVA model, adjusted for the baseline value of the outcome, was developed. Partial eta squared, and Cohen's d were used as effect sizes in ANCOVA and Independent t-test, respectively.

Cohen's d was calculated using the following formula [21]:

$$d=t\sqrt{\frac{1}{\mathrm{n}1}+\frac{1}{n2}}$$

With t = t statistic of the independent t-test (assuming homogeneity of variances) and n1 and n2 = the two sample sizes.

To calculate Cohen’s d in Mann–Whitney U test, we used an online calculator developed by Lenhard et al. [22]. The effect size were interpreted as small (d = 0.2), medium (d = 0.5), and large (d = 0.8) for Cohen’d and small (η2 = 0.01), medium (η2 = 0.06), and large (η2 = 0.14) for partial eta squared [21].

All statistical tests were two-tailed, and *p* values < 0.05 were considered significant. Statistical calculations were performed using the IBM SPSS Statistics for Windows, version 26 (IBM Corp., Armonk, N.Y., USA).

## Results

### Patient characteristic

**Fig. 1:**
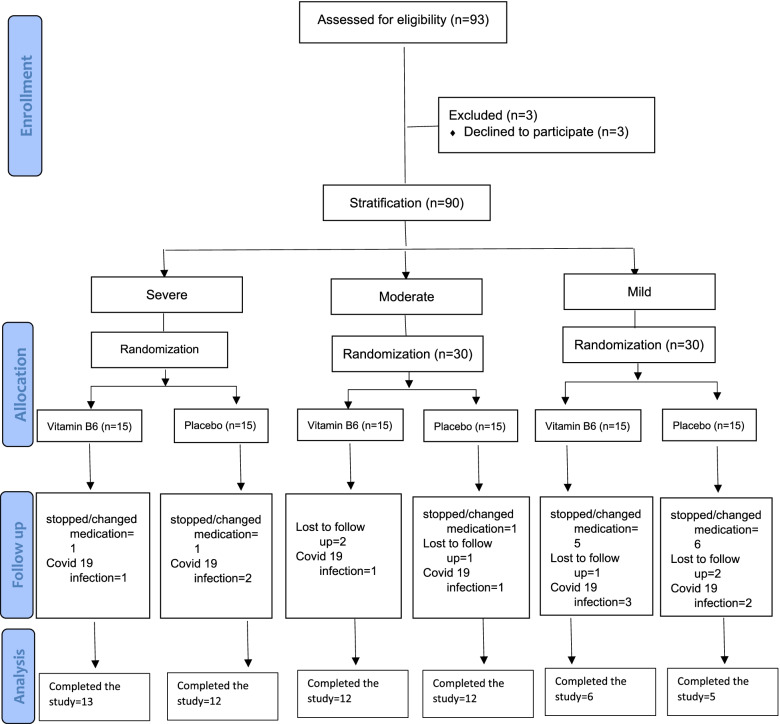
Flow diagram of participants

Of 90 eligible patients, 45 were randomized to the vitamin B6 group and 45 to the placebo group. 31 patients in the vitamin B6 group and 29 patients in the placebo group completed the study protocol and were recruited for the final analysis. The most common reason for exclusion in both study arms was non-adherence to the medications or taking over the counters (OTCs) containing vitamin B6 (Fig. 1). No adverse event was reported by participants. There was no statistically significant difference between the two groups regarding the baseline demographic and clinical characteristics. Table 1 shows the baseline characteristics of the participants in both groups. Tables 2 and 3 show the statistically non-significant difference in the baseline scores of disease severity, anxiety and depression, SF-12, pain-VAS, and laboratory values between the two groups (*p* value > 0.05).

**Table 1:** Baseline patient characteristics

Variable	Value		*P* value
	Vitamin B6 (*n* = 31)	Placebo (*n* = 29)	
Age, years (Mean (SD))	44.3 (8.0)	43.9 (11.4)	0.88
Education, years, number (percent)			
0–12	21 (67.7%)	20 (69.0%)	0.75
12–16	7 (22.6%)	8 (27.6%)	
> 16	3 (9.7%)	1 (3.4%)	
Marital status, number (percent)			
Single	4 (13.0%)	6 (20.7%)	0.50
Married	27 (87.0%)	23 (79.3%)	
Employment status, percent (number)			
Unemployed	22 (71.0%)	22 (75.9%)	0.77
Employed	9 (29.0%)	7 (24.1%)	
Time since diagnosis, years (median (SD))	2.0 (3.3)	1.0 (3.7)	0.32
Onset of symptoms, years (median (SD)	5.0 (6.0)	7.0 (6.9)	0.20
Prior medication, number (percent)			
Anti-depressant	28 (90.3%)	26 (89.6%)	1.0
Anti-epileptic	14 (45.1%)	12 (41.3%)	0.80
Analgesic	9 (29.0%)	6 (20.6%)	0.55

*SD* Standard deviation, *p* value < 0.05 is statistically significant

**Table 2:** Comparison of the clinical outcomes between the vitamin B6 and placebo groups

Variable	Vitamin B6		Placebo		*P* value	Effect size
	Baseline	After eight weeks	Baseline	After eight weeks		
FIQR-total						
Mean (SD)	54.66 (19.12)	43.47 (18.57)	51.79 (19.56)	39.77 (22.44)		
Adjusted mean (95%CI)		42.81 (36.1–49.4)		40.48 (33.6–47.3)	0.62^*^	0.004
Changes from baseline	-11.18 (19.78)		-12.02 (21.93)		0.87^**^	0.022
FIQR-function						
Mean (SD)	14.16 (7.00)	11.49 (6.28)	14.72 (7.21)	11.35 (8.03)		
Adjusted mean (95%CI)		11.62 (9.3–13.9)		11.21 (8.8–13.5)	0.80^*^	0.001
Changes from baseline	-2.66 (7.01)		-3.36 (7.53)		0.71^**^	0.096
FIQR-Overall						
Mean (SD)	10.41 (5.81)	7.96 (5.58)	10.79 (5.44)	7.93 (6.07)		
Adjusted mean (95%CI)		8.03 (6.0–10.0)		7.86 (5.8–9.9)	0.90^*^	< 0.001
Changes from baseline	-2.45 (6.69)		-2.86 (6.41)		0.81^**^	0.062
FIQR-symptom						
Mean (SD)	30.08 (8.77)	24.01 (9.18)	26.27 (10.09)	20.48 (9.87)		
Adjusted mean (95%CI)		23.12 (20.0–26.1)		21.43 (18.2–24.6)	0.45^*^	0.010
Changes from baseline	-6.06 (8.90)		-5.79 (10.39)		0.91^**^	0.028
Pain-VAS						
Mean (SD)	6.17 (1.97)	5.04 (2.27)	5.77 (2.60)	4.51 (2.46)		
Adjusted mean (95%CI)		4.98 (4.1–5.7)		4.58 (3.7–5.4)	0.49^*^	0.008
Changes from baseline	-1.12 (2.90)		-1.25 (2.48)		0.85^**^	0.048
HADS-anxiety						
Mean (SD)	10.96 (3.97)	9.25 (3.65)	9.00 (3.90)	7.96 (4.10)		
Adjusted mean (95%CI)		8.60 (7.5–9.6)		8.66 (7.6–9.7)	0.943^*^	< 0.001
Changes from baseline	-1.70 (3.17)		-1.03 (2.90)		0.39^**^	0.221
HADS-depression						
Mean (SD)	7.93 (3.87)	7.25 (3.38)	6.53 (3.17)	7.07 (3.98)		
Adjusted mean (95%CI)		6.73 (5.8–7.6)		7.64 (6.7–8.5)	0.16^*^	0.034
Changes from baseline	-0.67 (2.79)		0.53 (2.18)		0.209^***^	0.381
MCS of SF-12						
Mean (SD)	34.07 (11.74)	38.74 (11.87)	42.60 (15.52)	42.10 (15.37)		
Adjusted mean (95%CI)		40.71 (36.2–45.1)		39.40 (35.3–44.6)	0.82^*^	0.001
Changes from baseline	4.67		-0.49		0.157^**^	0.358
PCS of SF-12						
Mean (SD)	34.81 (8.87)	37.33 (10.14)	33.78 (11.38)	36.50 (11.95)		
Adjusted mean (95%CI)		37.04 (33.6–40.4)		36.81 (33.2–40.3)	0.92^*^	< 0.001
Changes from baseline	2.51 (10.38)		2.72 (10.22)		0.53^**^	0.019

*SD* Standard deviation, *CI* Confidence interval, *FIQR* Revised fibromyalgia impact questionnaire, *VAS* Visual analog scale, *HADS* Hospital anxiety depression scale, *SF12* Short-form health survey, *SF-12* 12-item short-form health survey, *MCS* Mental component summary, *PCS* Physical component summary

^*^ANCOVA, ** Independent t-test, *** Mann–Whitney U test, *p* value < 0.05 is statistically significant

**Table 3:** Comparison of the laboratory outcomes between the vitamin B 6 and placebo groups

Variable	Vitamin B12		Placebo		*P* value	Effect size
	Baseline	After eight weeks	Baseline	After eight weeks		
ESR						
Mean (SD)	14.32 (5.94)	13.52 (5.85)	14.65 (11.62)	14.25 (11.45)		
Adjusted mean (95%CI)		13.66 (12.0–15.2)		14.09 (12.4–15.7)	0.70^*^	0.003
Changes from baseline	-0.80 (3.55)		-0.40 (4.45)		0.73^**^	0.099
RDW						
Mean (SD)	12.65 (1.27)	12.86 (1.46)	12.94 (1.42)	12.91 (1.29)		
Adjusted mean (95%CI)		12.97 (12.7–13.1)		12.77 (12.5–13.0)	0.21^*^	0.032
Changes from baseline	0.19 (0.59)		-0.02 (0.52)		0.17^**^	0.387
PDW						
Mean (SD)	13.59 (2.13)	13.23 (1.69)	13.33 (2.25)	13.81 (2.63)		
Adjusted mean (95%CI)		13.10 (12.4–13.7)		13.94 (13.3–14.5)	0.06^*^	0.083
Changes from baseline	-0.43 (1.29)		0.48 (1.7)		0.05^**^	0.604
MPV						
Mean (SD)	10.55 (0.90)	10.53 (0.82)	10.39 (1.08)	10.26 (1.20)		
Adjusted mean (95%CI)	-	10.46 (10.1–10.7)	-	10.33 (10.0–10.6)	0.57^*^	0.008
Changes from baseline	-0.04 (0.65)		-0.12 (0.88)		0.74^**^	0.100
NLR						
Mean (SD)	1.80 (0.62)	1.73 (0.47)	1.69 (0.52)	1.70 (0.43)		
Adjusted mean (95%CI)	-	1.71 (1.5–1.8)	-	1.73 (1.5–1.8)	0.89^*^	< 0.001
Changes from baseline	-0.06 (0.55)		0.01 (0.46)		0.58^**^	0.157

*ESR* Erythrocyte sedimentation rate, *RDW* Red blood cell distribution width, *PDW* Platelet distribution width, *MPV* Mean platelet volume, *NLR* Neutrophil–lymphocyte ratio, *SD* Standard deviation, *CI* Confidence interval

^*^ANCOVA, ** Independent t-test, *** Mann–Whitney U test, *p* value < 0.05 is statistically significant. *p* value < 0.05 is statistically

### Comparison of the clinical outcomes between the vitamin B6 and placebo groups

Comparing the treatment effect on the disease severity in each group (intragroup comparison) showed a significant improvement in FIQR scores (total, function, overall, and symptom scores). The mean change of total FIQR was -11.18 ± 19.78 and -12.02 ± 21.93 in the vitamin B6 and placebo groups, respectively. However, there was no statistically significant difference in the score changes between the two groups (intergroup comparison) (*p* value = 0.87, d = 0.022). Although both groups showed a mild improvement in their pain-VAS scores after treatment (the mean of -1.12 ± 2.90 and -1.25 ± 2.48 score improvement in the vitamin B6 and placebo groups, respectively), the intergroup difference in pain-VAS was not statistically significant (*p* value = 0.85, d = 0.048).

Regarding psychological profile, patients in the vitamin B6 group and placebo showed an improvement in the mean scores of HADS-anxiety (-1.70 ± 3.17 and -1.03 ± 2.90 scores, respectively). However, the psychological improvement between the vitamin B6 and placebo groups was not statistically different (*P* value = 0.39, d = 0.221). Neither group showed any significant improvement in the HADS-depression score (-0.67 ± 2.79 Vs. 0.53 ± 2.18 in the vitamin B6 and placebo groups, respectively; *p* value = 0.20, d = 0.381).

There was no significant change in the inflammatory markers in both intragroup and intergroup comparisons. Using ANCOVA, adjusted for the baseline outcomes value, we showed no difference between the vitamin B6 and placebo groups in terms of disease severity, pain intensity, psychological profile, and inflammatory markers. Tables 2 and 3 show the detailed comparisons of the clinical and laboratory outcomes between the vitamin B6 and placebo groups (the results of the paired t-test are not shown in Tables 2 and 3).

## Discussion

This randomized double-blinded study showed that vitamin B6 (80 mg/day) had no superior effect to placebo in improving the pain, disease severity, psychological symptoms, health status, and inflammatory markers of the FM patients.

Given the role of vitamin B6 or pyridoxine on metabolic reactions, neurotransmitters balance, removing oxidative agents, and inflammation [4], it is crucial to clarify the potential effect of vitamin B6 on pain and psychological disturbance in FM. Theoretically, vitamin B6 supplementation can affect pain neurobiology and modify neurotransmitter imbalance toward increased pain threshold in the nociplastic pain conditions, including FM [9, 23]. It also may potentially modify the neuroplasticity changes occurring in psychological illness via its essential roles in the tryptophan/serotonin pathway, and catecholamines synthesis [24]. To the best of our knowledge, this is the first study evaluating the effect of vitamin B6 supplementation on FM impact and pain. The clinical studies dealing with vitamin B6 effect on pain conditions are scarce. Few previous studies showed the promising results of vitamin B6 effects on other nociplastic pain conditions such as low back pain and migraine headache [11]. Sadeghi et al. showed that 80 mg per day of pyridoxine attenuate the severity and duration of migraine headaches [12]. While some preclinical studies on rats showed the attenuated thalamic evoked nociceptive burst discharge and increased pain threshold, some clinical trials displayed that vitamin B6 monotherapy did not influence diabetic pain and neuropathy [13, 25, 26].

Although our results showed an improvement in pain, FM impact, HADS-anxiety score, and health status after vitamin B6 treatment, the same effect was also seen in the placebo arm of our participants. It must be noted that FM patients show a higher placebo response compared to other diseases. This effect has been reported even with FDA-approved medications for FM, which emphasizes the benefits of using placebo as dose extenders. It means that by pairing a placebo with physiologically active drugs, the medication dosage can be lowered without affecting the treatment response [27]. Considering this fact may warrant a larger sample size in future studies to display minimal differences in clinical outcomes between vitamin B6 treatment and placebo. In addition, we used 80 mg of vitamin B6 daily for two months which showed non-superiority to placebo. However, it is unclear whether a longer duration and higher dose of vitamin B6 treatment give a different outcome.

Although the theoretical benefit of vitamin B6 on psychological illness is compelling, our results didn't confirm this hypothesis. Existing systematic reviews have shown inconclusive results about the positive effects of vitamin B6 on emotional, cognitive, and mood disorders [28, 29]. The anxiety but not depression scores diminished during treatment with vitamin B6 in our FM patients, although this effect was also seen in the placebo group. We did not stop the antidepressant medications given to manage and control the FM symptoms due to ethical issues. Continuing the approved FM medications during our trial might attenuate the response rate to vitamin B6 treatment. Furthermore, it seems psychological improvement needs more prolonged treatment with a higher dose of vitamin B6.

It must be noted that FM is a heterogeneous nociplastic pain condition with a wide etiopathogenesis spectrum. It incorporates diverse comorbidities such as cognitive and psychological disturbance, and other comorbid nociplastic pain conditions such as headache, irritable bowel syndrome, and so on [30]. It would be conceivable that the therapeutic effect of one agent (such as vitamin B 6) may lead to different outcomes in the context of phenotype heterogeneity. Upon determining more homogenous FM subtypes/endotypes, it seems inevitable to continue the “try and error” approach to find the effective treatment for all heterogeneous subtypes. In the same way, we are unable to explain whether vitamin B6 therapy may result in different outcomes in different phenotypes of FM or whether the FM population in our study was representative of various phenotypes of this condition.

Furthermore, the previous studies on the effect of vitamin B6 supplementation in other types of pain have implied the controversial results [26]. They do not appear to be comparable with the results of our study in FM because the pain mechanisms in these conditions are different from FM etiopathogenesis and represent a more homogenous nature in each condition rather than FM. Therefore, the comparison of therapeutic response to vitamin B6 between FM and the other types of pain seems to be an oversimplification. More clarification of the vitamin B6 effect on the FM symptoms needs to include a larger FM population incorporating various phenotypes in future studies.

Furthermore, our study did not show any positive effect on the inflammatory markers after vitamin B6 treatment. Although several studies support the anti-inflammatory effect of vitamin B6 in different inflammatory conditions [31], this effect may be attenuated in the low-grade or central inflammation situations such as FM. The purported neuroinflammation and low-grade systemic inflammation in FM may not be detectable by usual methods. We used NLR, PDW, MPV, ESR and RDW as feasible and cost–benefit markers to assess the anti-inflammatory role of vitamin B6 in FM. While these markers were previously introduced as inflammatory biomarkers in FM and other inflammatory diseases [6, 7, 32, 33], they seem non-sensitive methods to detect minimal inflammation or changes in FM during non-potent anti-inflammatory treatment such as vitamin B6.

Our study had some limitations. First, we recruited only female participants from one tertiary center; thus, the study findings cannot be extrapolated to the general FM population. Second, we used a single dose of vitamin B6 as adjuvant therapy; Trial studies with different dosages of vitamin B6 in naïve patients may lead to different results. Third, given the nature of the large placebo effect in FM patients, it would be desirable to evaluate the effect of vitamin B6 in a larger population size of control studies compared with placebo and FM FDA-approved drugs. Although designing such studies may be difficult from point of practical perspective, it can clarify the effects of vitamin B6 on pain and psychological impacts of FM patients.

Vitamin B6 mechanistically shows a potential role in modifying pain and psychological disturbance in nociplastic pain, including FM. While the clinical and preclinical data on this issue is contradictory, the positive effects of vitamin B6 on the mentioned outcomes have been promising in a different situation. This study aimed to shed more light on the vague effects of vitamin B6 treatment in FM as a disputing condition in the management. Acknowledgment of vitamin B6 as a relatively safe adjuvant treatment needs future and larger controlled studies.

## Data Availability

The datasets generated during and analyzed during the current study are not publicly available due to confidentiality but are available from the corresponding author upon reasonable request.
